# Volatile Constituents from *Catasetum* (Orchidaceae) Species with Occurrence in the Brazilian Amazon

**DOI:** 10.3390/plants12040703

**Published:** 2023-02-05

**Authors:** Franciléia M. de Vasconcelos, Eloisa Helena A. Andrade, Luiz Otávio A. Teixeira, Pablo Luis B. Figueiredo, José Guilherme S. Maia

**Affiliations:** 1 Programa de Pós-Graduação em Química, Universidade Federal do Pará, Belém 66075-110, PA, Brazil; 2 Associação dos Orquidófilos do Pará, Parque da Residência, Belém 66063-240, PA, Brazil; 3 Departamento de Ciências Naturais, Centro de Ciências Sociais e Educação, Universidade do Estado do Pará, Belém 66050-540, PA, Brazil; 4 Programa de Pós-Graduação em Química, Universidade Federal do Maranhão, São Luís 65085-580, MA, Brazil

**Keywords:** floral scents, olfactory signals, principal component and hierarchical cluster analyses, *trans*-geranylgeraniol, 1,4-dimethoxybenzene, linalool, 2-phenylethyl acetate, geraniol, 7-*epi*-1,2-dehydro-sesquicineole, benzyl acetate

## Abstract

Background: *Catasetum* Rich. ex Kunth is a genus of Neotropical orchids distributed in Central and South American regions. In the Brazilian Amazon, there are more than 60 species of *Catasetum*. The floral aromas of orchids are little known, particularly of *Catasetum* species. This work aimed to analyze the chemical constituents of the volatile concentrates of eight *Catasetum* specimens from the Amazon: *C. alatum* (1), *C. albovirens* (2), *C. barbatum* (1), *C. ciliatum* (2), *C. galeritum* (1), and *C. gnomus* (1). Methods: Gas chromatography (GC) and gas chromatography-mass spectrometry (GC-MS) analyzed and identified the constituents of the volatile concentrates, and principal component analysis (PCA) and hierarchical cluster analysis (HCA) were used in the multivariate statistical analysis. Results: The *Catasetum* main constituents in descending order and above 10% were *trans*-geranylgeraniol, 1,4-dimethoxybenzene, linalool, 2-phenylethyl acetate, geraniol, 7-*epi*-1,2-dehydro-sesquicineole, 1,8-cineole, benzyl acetate, limonene, methyl salicylate, (*E*)-β-farnesene, anisyl butyrate, *cis*-carvone oxide, cadin-4-en-10-ol, indole, α-pinene, and δ-cadinene. Conclusions: Multivariate statistical analysis of *Catasetum* species showed that *C. barbatum*, *C. albovirens*, and *C. gnomus* are distinct from the other studied species, while *C. alatum*, *C. ciliatum*, and *C. galeritum* presented the same primary classes of compounds. These results contribute to a better understanding of the genus *Catasetum* chemotaxonomy.

## 1. Introduction

Flowering plants and their volatile compounds attract birds, insects, and other animals, including mammals, as pollinators for their reproduction. More than 1700 flower scent compounds, covering 990 taxa, have already been identified [1,2,3]. The flower perianth is primarily responsible for the scent emission, though all floral organs might contribute to the emission of scents. Floral scents are stored in the oily glands, such as the trichomes before it is released into the air as volatile compounds, and in addition to flowers, volatile compounds emitted by other plant organs have involved in its defense mechanisms. Therefore, floral volatiles play a significant role in the plant’s reproductive process by attracting pollinators and acting as repellents and physiological protectors against abiotic stress [2,4]. Floral volatiles are expected to be used in the composition of perfumes, cosmetics, flavors, and therapeutic applications. However, the volatiles emitted by flowers are also the main signals captured by insects to select gratifying flower species associated with the respective flower colors [3,5]. Floral scents are composed of volatile compounds, usually lipophilic and low molecular weight. Based on their origin, function, and biosynthesis, floral scents are grouped into three main classes of compounds: Terpenoids, phenylpropanoids/benzenoids, and fatty acid and derivatives [3,6].

Orchidaceae is the most prominent flowering plant family, with 736 genera and ca 28,000 species, which shows a wide diversity of epiphytic and terrestrial specimens, colonizing almost every earth’s habitat and renowned for their abundance of morphological types, with an unending number of beautiful variations and very well represented in the monocotyledons’ floral evolution [7,8]. The orchids distributed in the natural environment have small sizes and have a limited production of flowers. These restrictions are circumvented by producing visual and olfactory signals to attract pollinators. Developing highly specialized pollination mechanisms to attract effective pollinators is a common strategy in orchids. Specializing these mechanisms leads to the formation of syndromes, in which the set of floral characteristics, including fragrances, is associated with attracting a particular group of pollinators. Euglossini syndrome is widely known among orchids and is characterized by the absence of food resources, with only the production of specialized floral fragrances collected by male Euglossini bees. Floral fragrances not only act as signaling but also represent a reward for bees because some components of these mixtures will compose their pheromones, which is the essential prerequisite for sexual recognition and selection during mating [9,10,11,12].

*Catasetum* Rich. *ex* Kunth is one of the genera of Neotropical orchids pollinated by male Euglossini bees. The genus has about 130 species distributed mainly in Central and South American tropical regions. Floral fragrances play a vital role in the diversification of *Catasetum*. Hybridizations that result in changes in the composition of fragrances also generate differences in pollinators, restricting gene flow and contributing to the origin of new strains. Inflorescences of *Catasetum* are usually unisexual with distinct morphology and sexual determination according to each individual’s environmental and nutritional characteristics. *Catasetum* usually shows male or female flowers, but in some situations, may form nonfunctional hermaphroditic flowers and/or flowers of both sexes. Sex expression is controlled by plant size and light intensity. Large plants under strong sunlight usually develop female flowers, whereas younger and smaller plants under moderate light develop male flowers. They are sympatric species that use floral fragrances as a fundamental part of the reproductive isolation mechanism. The composition of these aromas is represented by a mixture of constituents with attractive and repellent action of different proportions, detected in small amounts by male Euglossini bees. Therefore, orchids attract particular species of bees that, in synergy with other floral filters (e.g., morphological characteristics), act as their effective pollinators. [13,14,15,16].

The present work aimed to extract the volatile concentrates and identify the chemical constituents of the flowers of eight specimens from six species of *Catasetum*: *C. alatum* (1), *C. albovirens* (2), *C. barbatum* (1), *C. ciliatum* (2), *C. galeritum* (1), and *C. gnomus* (1), with occurrence in the Brazilian Amazon. In addition, submit the chemical composition data of these specimens to multivariate analysis, targeting their association with other species/specimens of *Catasetum* taxonomically close or previously analyzed.

## 2. Results and Discussion

### 2.1. Catasetum Rich. ex Kunth

#### 2.1.1. *Catasetum alatum* M.F.F.Silva & A.T.Oliveira

**Figure 1:**
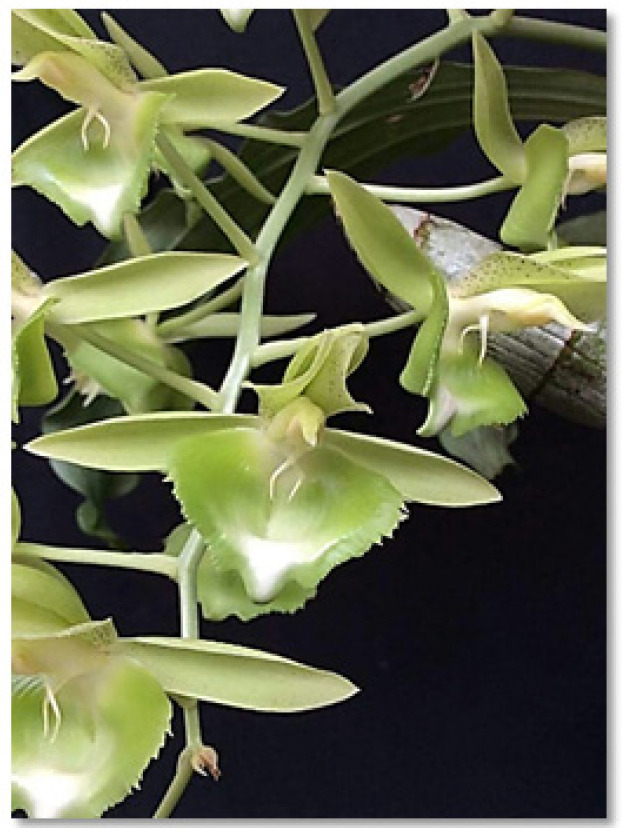
*Catasetum alatum.* (Source Luiz Otavio Adão).

**Botanical description:** Epiphyte. Pseudobulbs aggregated, multi-ringed, fusiform, erect; apex abruptly acuminate. Leaves membranous, lanceolate, slightly wavy margins, five to eight leaves per pseudobulb, and three thin veins. Inflorescence 10 to 20 flowers (Figure 1), basal and pendant. Flowers staminate, resupinate jade green, erect, and distribute in the rachis’s middle third. Dorsal sepal lanceolate, erect, slightly concave; lateral sepals erect, linear-lanceolate, green, arched backward, acuminate. Petals oblong-lanceolate, greenish, convex, erect, margins slightly serrated. Fleshy lip, jade-green, 90° angle with the spine; frontal opening or elliptical ostium, internally light green, white spot at base, sack form at middle portion; edges of lateral lobes strongly winged, semi-curled, slightly serrated, asymmetrical, raised; triangular terminal lobe, apiculate, facing downwards, edges smooth [17]. Flowering in December and January.

**Geographic distribution:** Endemic in Brazil. Occurrence for the North region of Brazil, State of Rondônia, in riparian or gallery forest areas [17].

The specimen of *C. alatum* in this study was sampled initially in the locality of Vila Nova California, Rondônia, Brazil. Table 1 lists the constituents of its volatile concentrate.

**Figure 2:**
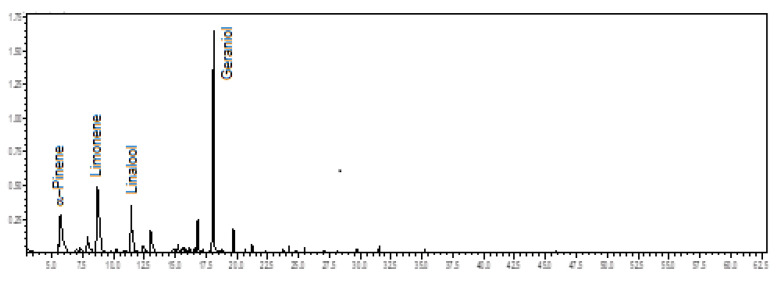
Ion-chromatogram of the *Catasetum alatum* volatile concentrate.

Oxygenated monoterpenes (57.0%) predominated in the volatile concentrate of *C. alatum*, followed by monoterpene hydrocarbons (36.9%). The main constituents were geraniol (29.7%), limonene (18.2%), α-pinene (11.8%), and linalool (8.8%) (see Figure 2). The floral scents of *C. alatum* are being described for the first time.

#### 2.1.2. *Catasetum Albovirens* Barb. Rodr.

**Figure 3:**
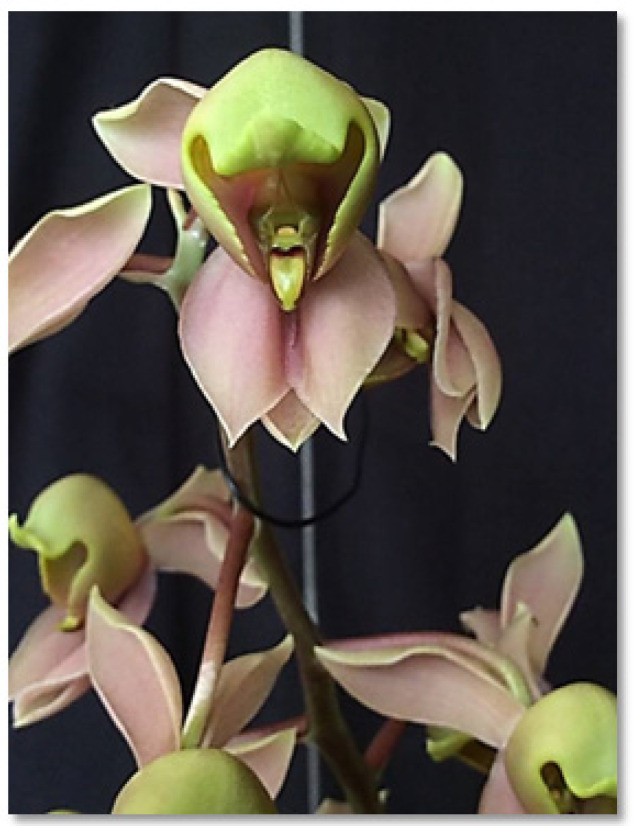
*Catasetum albovirens.* (Source Luiz Otavio Adão).

**Botanical description:** Epiphyte. Fusiform pseudobulbs. Leaves with longitudinal veins, elliptical-lanceolate, sharp at the base, attenuated in canaliculate pseudo petiole. Inflorescence about 25.0 cm, erect and at middle curved in a basal arch, multiflora. Flowers (Figure 3) not resupinate, white-green to pink-green, drooping. Pedicel patent and twisted. Oval or oval-oblong sepals, obtuse or very sharp; erect dorsal sepal with five prominent veins; oblique lateral sepals. Petals lanceolate and acuminate with three very distinct veins. Lip nearly globular, distinctly 3-lobed, crass-fleshy, generally greener than other segments; lateral lobes rounded, entire, curved; terminal lobe patent, reflex, more or less 3-lobed or truncated, glabrous and smooth on the inside. Oval column, with rostellum, acuminate and straight. Antennae about 6.0 mm, extending to the front [17]. Flowering in February to May.

**Synonimy:***Catasetum lamosii* Rolfe [17].

**Geographic distribution:** Endemic in Brazil, occurring in the states of Amazonas, Pará, Maranhão, Mato Grosso, and Tocantins, in anthropic areas such as rupestrian fields, high and flooded forests, seasonal deciduous forests, and savannas [17].

Two specimens of *C. albovirens* were analyzed. Specimen Calb-1 was sampled initially in the municipality of Muaná, Ilha do Marajó, and specimen Calb-2 was collected initially in the municipality of Tucuruí, Pará state, Brazil. Table 2 lists the constituents of their volatile concentrates.

**Figure 4:**
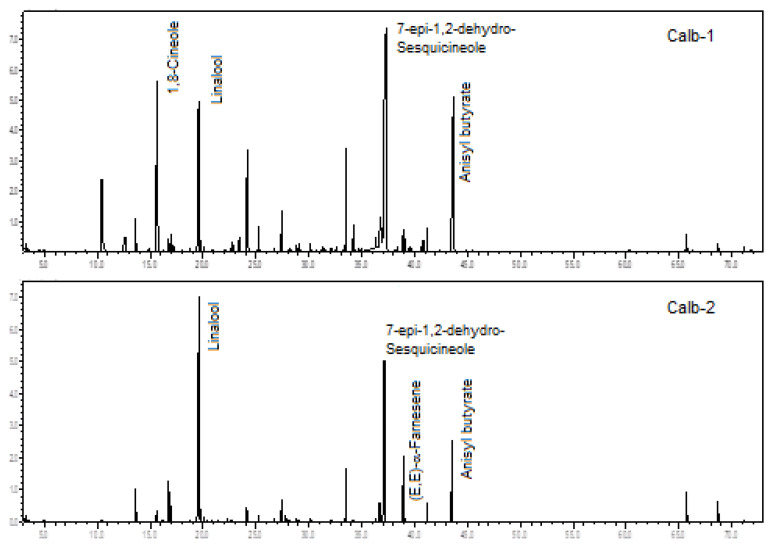
Ion-chromatogram of the *Catasetum albovirens* volatile concentrates.

The two analyzed specimens of *C. albovirens* were rich in oxygenated monoterpenes (52.4% and 36.7%), oxygenated sesquiterpenes (30.4% and 20.8%), and benzenoids (7.5% to 15.4%). There was a predominance of linalool (10.0% to 39.5%), 7-*epi*-1,2-dehydro-sesquicineole (19.3% to 28.3%), anisyl butyrate (7.5% to 15.4%), 1,8-cineol (1.1% to 11.7%), and (*E*,*E*)-α-farnesene (1.0% to 5.2%), analyzing both specimens (see Figure 4).

A previous comparative biology study of *C. albovirens* showed β-myrcene, eucalyptol (1,8-cineole), (*E*)-β-ocimene, linalool, 2,4-dimethylacetophenone, indole, (*E*)-8-hydroxy-linalool, methyl anthranilate, geranyl acetate, and (*E*,*E*)-farnesene, as primary constituents [18]. Therefore, presenting some chemical similarities to the two samples currently analyzed.

#### 2.1.3. *Catasetum barbatum* Lindl.

**Figure 5:**
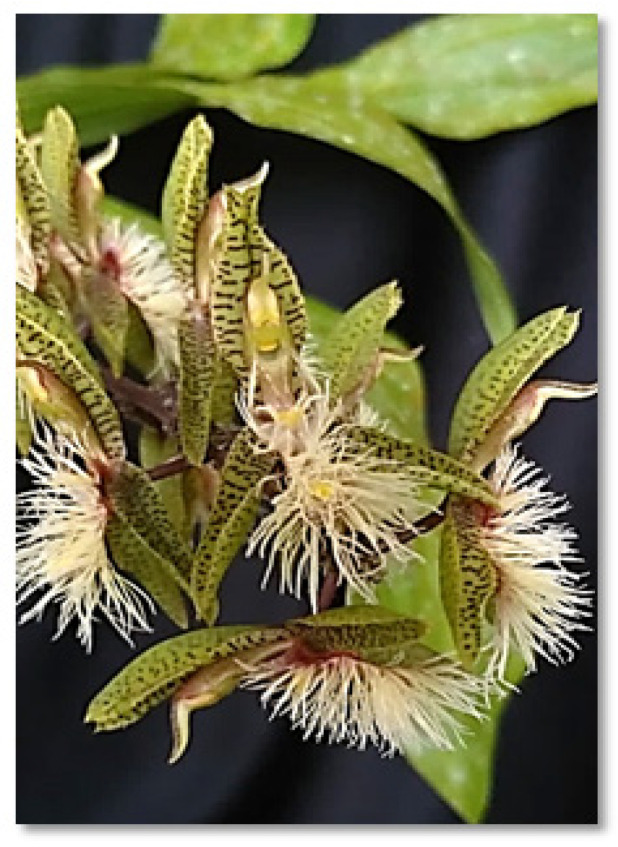
*Catasetum barbatum.* (Source Luiz Otavio Adão).

**Botanical description:** Epiphytic, occasionally terrestrial. Fusiform pseudobulbs, about 15.0 to 5.0 × 3.0 to 5.0 cm long. Narrow, plicate leaves with a midrib and two lateral ones. Inflorescence suberect or arched with up to 20 flowers. Flowers (Figure 5) male resupinate, greenish with brown spots. Sepals are lanceolate to dorsal erect, the lateral ones reflexed on the pedicel. Petals lanceolate and erect, margins finely serrated, somewhat revolute. The lips include hairs that may be white or greenish; the basal callus may be simple or bifurcated, usually surrounded by pilosity. Column light green or brownish. Cream anther. Yellow pollinia are hard and compressed on a white laminar stipe and a white viscid disc. Flowering in April and May [17].

**Synonimy:***Catasetum barbatum* var. *spinosum* Rolfe, *C. brachybulbon* Schltr., *C. buchtienii* Kraenzl., *C. comosum* Cogn., *C. crinitum* Linden, *C. polydactylon* Schltr., *C. proboscideum* Lindl., *C. rionegrense* Campacci & G.F. Carr, *C. spinosum* (Hook.) Lindl., *C. variabile* Barb. Rodr., *Myanthus barbatus* Lindl., *M. barbatus* var. *immaculatus* Knowles and Westc., *M. spinosus* Hook. [17].

**Geographic distribution:** Not endemic in Brazil, but found in upland and floodplain forests, broadleaf forests, mangrove and palm groves, and vegetation on rocky outcrops in the states of Amazonas, Pará, Roraima, Tocantins, Alagoas, Bahia, Ceará, Maranhão, Paraíba, Pernambuco, Piauí, Distrito Federal, Goiás, Mato Grosso do Sul, Mato Grosso, and Minas Gerais [17].

The specimen of *C. barbatum* in this study was sampled initially in the municipality of Ourilândia do Norte, Pará, Brazil. Table 3 lists the constituents of its volatile concentrate.

**Figure 6:**
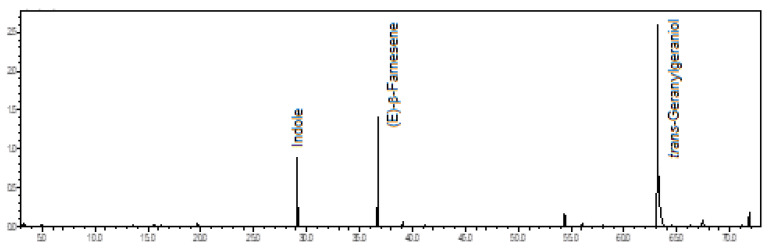
Ion-chromatogram of the *Catasetum barbatum* volatile concentrate.

In this volatile concentrate of *C. barbatum* flowers, the major constituents were the *trans*-geranylgeraniol (61.2%), an oxygenated diterpene, the (*E*)-β-farnesene (16.4%), a sesquiterpene hydrocarbon, and the indole (11.3%), a heterocyclic aromatic compound (see Figure 6).

The floral scents of *C. barbatum* were previously analyzed. Two morphological variants originated from Brazil and Ecuador has differed in fragrance. The Brazilian form showed as primary constituents 1,8-cineole (16.1%), a-pinene (7.9%), and an unidentified main oxygenated monoterpene (46.1%), while the Ecuadorian form displayed ocimene (67.7%) as the main compound [19]. Also, another paper on *C. barbatum* floral scent presented germacrane-type compounds as the primary constituents, including germacra-1(10)-5-dien-4-ol (60.0%), germacrene A (9.0%), germacrene D (7.0%), and bicyclogermacrene (2.0%) [16]. A doctoral thesis in the comparative biology of *Catasetum* presented (*Z*)-α-bergamotene, neryl acetate, and (*E*)-β-farnesene as the main components of *C. barbatum* sampled in the Amazonas state, Brazil [18]. This last sample of *C. barbatum* is more associated with the one analyzed in the present work due to the presence of (*E*)-β-farnesene, except for the significant content of *trans*-geranylgeraniol in the sample worked by us.

#### 2.1.4. *Catasetum ciliatum* Barb. Rodr.

**Figure 7:**
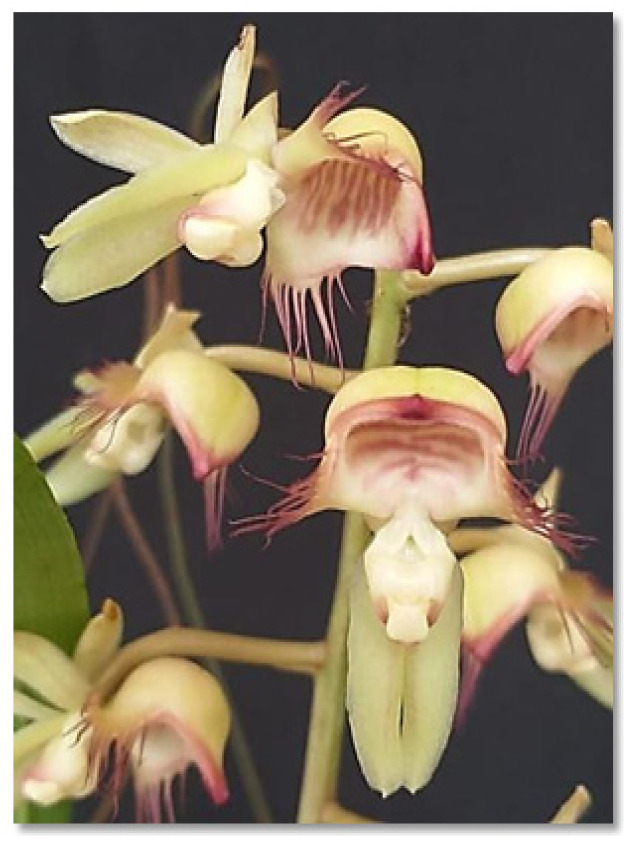
*Catasetum ciliatum*. (Source Luiz Otavio Adão).

**Botanical description:** Epiphytic, erect, caespitose, ca. 38.0 cm long. Short rhizome, less than 1.0 cm between pseudobulbs. Caulomas thickened into pseudobulbs, aggregated, fusiform covered by leaf sheaths. Elliptical, semi-leathery leaves, five prominent veins. Inflorescence in a raceme, lateral; green peduncle, partially covered by tubular sheaths, ca. six flowers. Flowers (Figure 7) resupinate, greenish; sepals free from each other, dorsal elliptical; apex acuminate, elliptical-falcated laterals, apex acuminate; slightly oval petals. Lip green, elm-shaped, narrow base, adnate at the foot of the gynostemium, apex narrowly curved. Flowering in April and May [20].

**Geographic distribution:** Not endemic In Brazil but found in areas of campinarana, rock fields, floodplain forests, sandbanks, and savannas, in the states of Amazonas, Amapá, Pará, Rondônia, Roraima, and Maranhão.

Two specimens of *C. ciliatum* were analyzed. Specimen Ccil-1 was sampled initially in the municipality of Prainha, and specimen Ccil-2 was collected initially in the locality of Lago Preto, municipality of Santarém, Pará state Brazil. Table 4 lists the constituents of their volatile concentrates.

**Figure 8:**
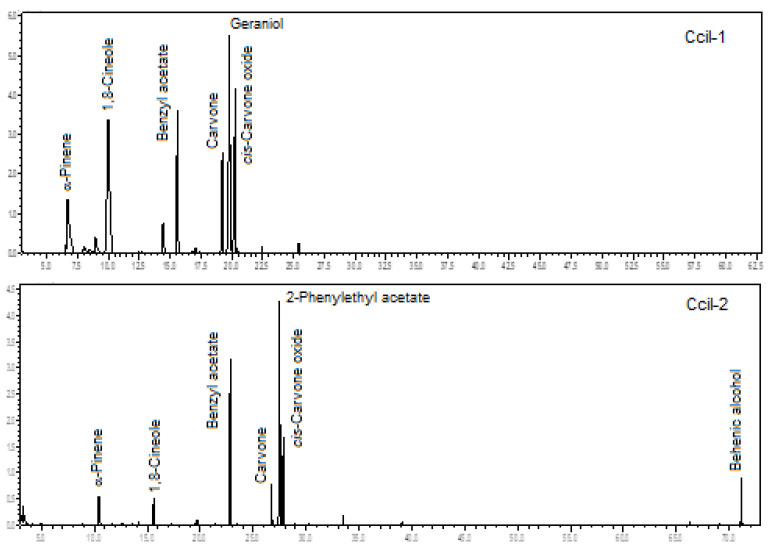
Ion-chromatogram of the *Catasetum ciliatum* volatile concentrates.

The two analyzed specimens of *C. ciliatum* were rich in oxygenated monoterpenes (67.5% and 24.6%), benzenoids/phenylpropanoids (15.0% and 53.5%), and monoterpene hydrocarbons (14.8% and 8.4%). There was a predominance of 2-phenylethyl acetate (3.2% to 30.5%), 1,8-cineole (3.7% to 23.7%), benzyl acetate (11.8% to 22.8%), geraniol (5.0% to 16.6%), *cis*-carvone oxide (10.1% to 14.3%), α-pinene (4.8% to 10.5%), and carvone (4.4% to 7.8%), analyzing both specimens (see Figure 8). The floral scents of *C. ciliatum* are being described for the first time.

#### 2.1.5. *Catasetum galeritum* Rchb.f.

**Figure 9:**
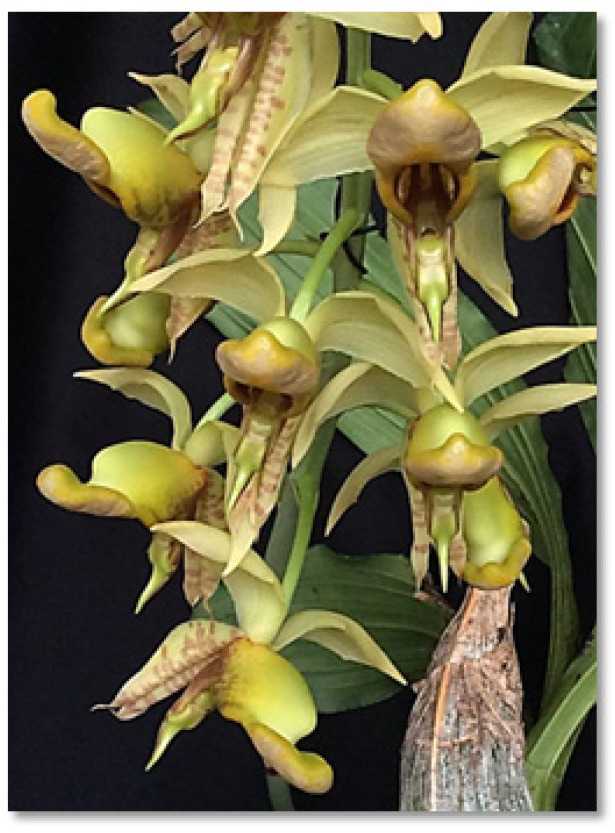
*Catasetum galeritum.* (Source Luiz Otavio Adão).

**Botanical description:** Epiphyte. Conical-fusiform pseudobulbs, compressed on the sides, ringed and furrowed. Leaves lanceolate-spatulate, acuminate towards the base, attenuated in canaliculate pseudo petiole, with five to seven longitudinal veins. Inflorescence from 20.0 to 25.0 cm, racemose, basal, pendant, and robust. Pedicel patent, arched, plump with pseudo-ovary. Flowers (Figure 9) are not resupinate and patent. Petals oblong-ligulate, sharp, erect, conniving with the dorsal sepal and embraced by its margins. Sepals are slightly convex and attenuated at the base. Very patent lip, somewhat reflexive, inferior, thickly fleshy, rigid, longer than the lateral sepals, with an oblong sack form protuberance, laterally compressed, entire, the sides erect over the sac projected forward. Fleshy column, elongated, at apex short conical rostrum, slightly curved. Antennae about 18.0 mm long, parallel, converging, with two yellow pollinia. The flowers of *C. galeritum* release an intense and sweet perfume, easily detectable by the human nose up to 2.0 m away [21]. Flowering in April.

**Synonimy:***Catasetum galeritum* var. *pachyglossum* Rchb.f. [17].

**Geographic distribution:** Endemic in Brazil, occurs in riparian or gallery forests, Upland and floodplain forests, and broadleaf forests in the states of Amazonas, Pará, Tocantins, Maranhão, and Mato Grosso [17].

This *C. galeritum* specimen was sampled initially in the municipality of São Félix do Xingu, Pará state, Brazil, and the constituents of their volatile concentrate are listed in Table 5.

**Figure 10:**
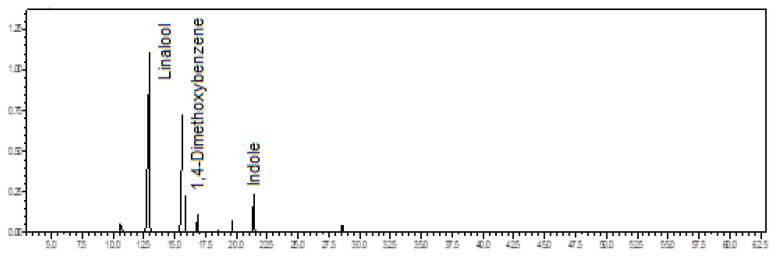
Ion-chromatogram of the *Catasetum galeritum* volatile concentrate.

Benzenoids/phenylpropanoids (59.3%) predominated in the volatile concentrate of *C. galeritum*, followed by oxygenated monoterpenes (37.6%). The main constituents were 1,4-dimethoxybenzene (54.1%), linalool (34.9%), and indole (5.2%) (see Figure 10). A previous floral scent of another *C. galeritum* specimen, also occurring in the municipality of São Felix do Xingu, Pará state, Brazil, and visited only by male bees, was similarly composed of 1,4-dimethoxybenzene (85-94%), followed by minor amounts of linalool, indole, β-elemene, and (*E*)-caryophyllene [21].

#### 2.1.6. *Catasetum gnomus* L. Linden & Rchb.f.

**Figure 11:**
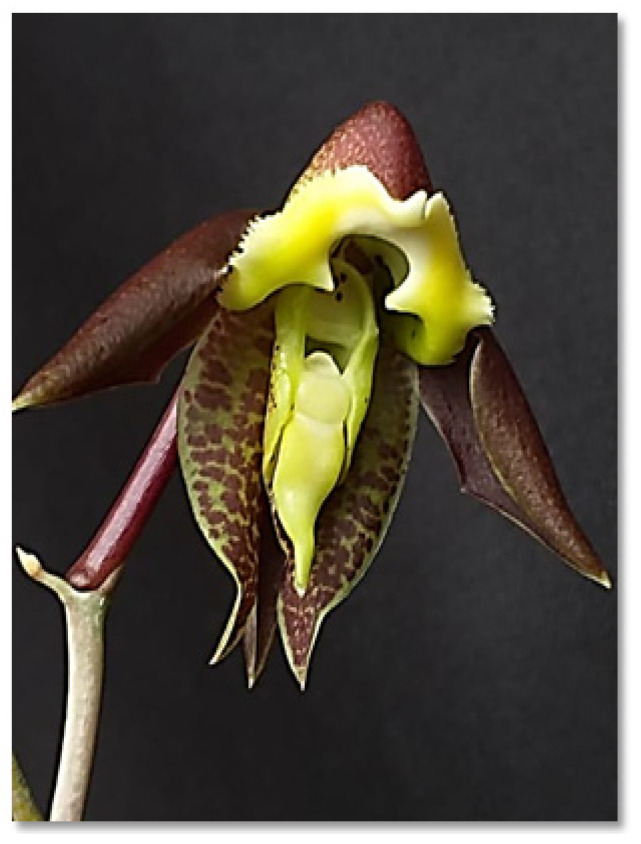
*Catasetum gnomus.* (Source Luiz Otavio Adão).

**Botanical description:** Epiphyte. Pseudobulbs fusiform, erect, slightly compressed on the sides, long attenuated and acuminate, aggregated. Leaves oblong-lanceolate, 7 to 9 per pseudobulb, 3-veined, erect-patent. Inflorescence male basal, racemose, arched to pending. Bracts amplexicaul lanceolate, acuminate. Flowers (Figure 11) 5 to 15 non-resupinate, alternate on the rachis, somewhat inclined or drooping. Triangular floral bracts run to the pedicels. Pedicel cylindrical, sinuous. Dorsal sepal oboval-lanceolate, attenuated base, acuminate and acute apex; the sides oboval-lanceolate, slightly united at the base, apex very acuminate. Petals linear-lanceolate, erect, partially covered by the dorsal sepal and covering the column, oblique. Fleshy lip, with sack form protuberance (25.0 mm bristle), undulating and crenulated margins, sometimes serrated, internally under the column with a carina and around the thickened ostium, the anterior side always with the margins reclined downwards. Fleshy column, erect, triangular in cross-section, apex rostriform, threadlike, and long. Crossed antennas. Two yellow pollinia. Anther yellowish [17]. Flowering in February.

**Synonimy:***Catasetum gnomus* var. *phasma* (Rchb.f.) Cogn., *Catasetum heteranthum* Barb. Rodr., *Catasetum huebneri* Mansf., *Catasetum mocuranum* Schltr., *Catasetum negrense* Schltr., *Catasetum phasma* Rchb.f.

**Geographic distribution:** Endemic in Brazil occurs in riparian or gallery forests, Upland and floodplain forests, and broadleaf forests in the states of Amazonas, Pará, and Rondônia.

This *C. gnomus* specimen was sampled initially in Manaus, Amazonas state, Brazil, and the constituents of its volatile concentrate are listed in Table 6.

**Figure 12:**
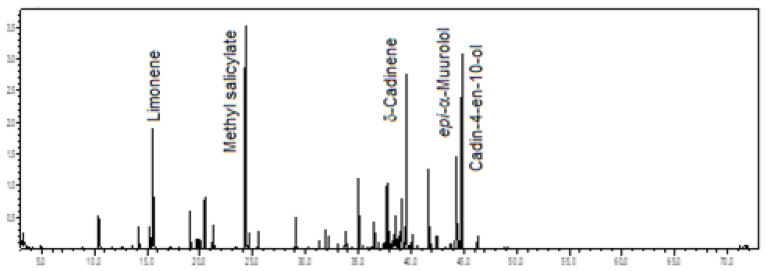
Ion-chromatogram of the *Catasetum gnomus* volatile concentrate.

Sesquiterpene hydrocarbons (29.0%) predominated in the volatile concentrate of *C. gnomus*, followed by oxygenated sesquiterpenes (26.3%), benzenoids/phenylpropanoids (21.4%), monoterpene hydrocarbons (13.2%), and oxygenated monoterpenes (8.5%). The main constituents were methyl salicylate (17.0%), cadin-4-en-10-ol (12.1%), δ-cadinene (10.3%), limonene (7.5%), and epi-α-muurolol (6.9%) (see Figure 12). The floral scents of two other *C. gnomus* specimens grown in a greenhouse at the University of Miami, Florida, USA, were previously reported, showing methyl salicylate, α-pinene, 1,8-cineole, and methyl benzoate as their main constituents [16,19,22].

### 2.2. The Catasetum Floral Scent Chemistry

The primary constituents found in the *Catasetum* flowers analyzed in this work were the monoterpenes linalool, 1,8-cineole, α-pinene, limonene, geraniol, carvone, and *cis*-carvone oxide; the sesquiterpenes 7-*epi*-1,2-dehydro-sesquicineole, (*E*,*E*)-farnesene, (*E*)-β-farnesene, δ-cadinene, *epi*-α-muurolol, and cadin-4-en-10-ol; the benzenoids anisyl butyrate, indole, benzyl acetate, 2-phenylethyl acetate, methyl salicylate, and 1,4-dimethoxybenzene; and the diterpene *trans*-geranylgeraniol. By analogy, consulting the literature, it was found that the floral aromas of 30 species of *Catasetum* (around 17% of existing) have already been chemically characterized. In these studies, 124 volatile compounds were reported, belonging to the following classes of compounds: monoterpenes (44), sesquiterpenes (26), irregular terpenes (1), aliphatics (14), aromatics (38), and N-bearing compounds (1). Individually, 1,8-cineole and α-pinene were the most reported, followed by β-pinene, (*E*)-dihydrocarvone, (*E*)-carvone epoxide, carvone, and *p*-cymene (10) [2,16].

The complexity of floral scents in the species of *Catasetum* investigated so far varies considerably. In the present work, more than 93% of the scent profile has been characterized, while the number of identified constituents varied from 1 in *C. micranthum* to 74 in *C. uncatum* [23,24]. This variation in scent floral complexity across *Catasetum* species certainly reflects an inherent characteristic for each species. As expected in angiosperms, floral scents of *Catasetum* are species-specific, although dominated by some significant constituents usually shared by several species [25]. Most *Catasetum* species have 2 or 3 main constituents that account for more than 70% of the fragrances. These constituents are potent attractants to many *Euglossa* and *Eulaema* bees, but the attractiveness to individuals and species is reduced as more components compose the mixtures so that specific scents attract only a few pollinator species [11,19].

The pivotal role of floral fragrances in pollinator shifts and as a reproductive isolating mechanism in *Catasetum* was previously highlighted [16]. However, floral scents may not be enough to assure the effective reproductive isolation in *Catasetum*. Sympatric species usually produce similar fragrances, thereby attracting the same pollinator species. In these cases, different reproductive isolating mechanisms (e.g., geographical, morphological/mechanical, temporal/seasonal), acting alone or together, will be necessary to contribute to or prevent the hybridization [16,19]. Presently, considering the well-defined separation of the pollinating genera of *Catasetum* and the higher sensorial similarity between the closely related bee species, have been speculated that the olfactory adaptations have shaped the evolution of floral fragrances of *Catasetum* due to the partitioning with pollinator’s bees, particularly from the genera *Euglossa* and *Eulaema* [16,19].

Therefore, pollinator affinity with phylogeny is correlated with differences found in floral aromas. More generally, the question is why flowers produce different odors or why mixtures of odors tend to be species-specific. The answer to this question demands more complex functional analyses, attributing phylogenetic, physiological, and ecological influences to the chemical variation of floral scents [25].

### 2.3. Catasetum Specimens’ Multivariate Analysis

The floral variability of samples of *Catasetum* volatile concentrates was evaluated using multivariate statistical analyses (PCA, principal component analysis; HCA, hierarchical cluster analysis) based on their classes of compounds. The percentage values of monoterpene hydrocarbons (MH), oxygenated monoterpenes (OM), sesquiterpene hydrocarbons (SH), oxygenated sesquiterpenes (OS), oxygenated diterpenes (OD), benzenoids/phenylpropanoids (B/P), and fatty acids and derivatives were obtained based on the GC-MS analyses of the volatile concentrate constituents. The data were used as variables (see Table 7).

**Figure 13:**
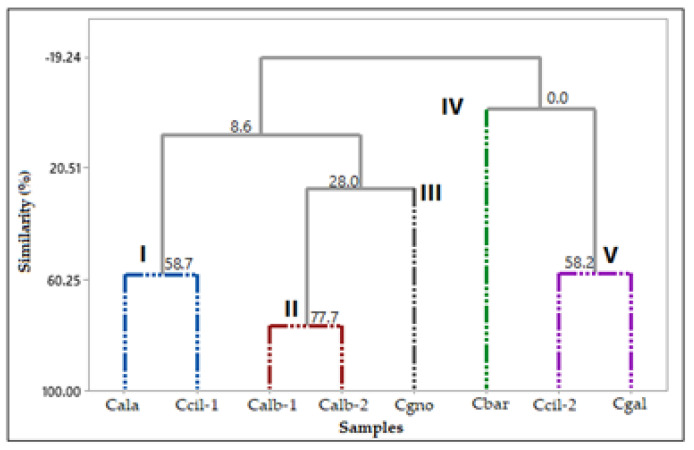
Hierarchical cluster analysis (HCA) of the *Catasetum* volatile concentrates, based on their classes of compounds.

The HCA analysis (Figure 13) showed the formation of five groups. The first group comprised *C. alatum* and *C. ciliatum-1* (I); the second group the two specimens of *C. albovirens* (II); the third group of *C. gnomus* (III); the fourth group of *C. barbatum* (IV); and fifth group by *C. ciliatum-2* and *C. galeritum* (V).

**Figure 14:**
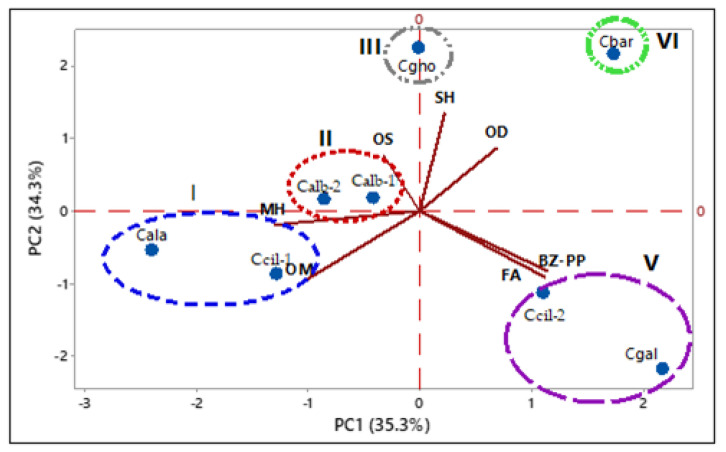
Principal componente analysis (PCA) of the *Catasetum* volatile concentrates, Based on their classes of compounds.

The PCA analysis (Figure 14) explained 69.5% of the data variability. The PC1 justified 35.32% of the data, showing negative correlations with monoterpene hydrocarbons (MH, λ = −0.52), oxygenated monoterpenes (OM, λ = −0.43), oxygenated sesquiterpenes (OS, λ = −0.12) and positive correlations with sesquiterpene hydrocarbons (SH, λ = 0.09), oxygenated diterpene (OD, λ = 0.28), benzenoids/phenylpropanoids (BZ-PP, λ = 0.46), and fatty acids and derivatives (FA, λ = 0.45). The PC2 clarified 34.3% of the data, showing a positive correlation with oxygenated sesquiterpenes (OS, λ = 0.32), oxygenated diterpene (OD, λ = 0.36), and sesquiterpene hydrocarbons (SH, λ = 0.56), and negative correlation with monoterpene hydrocarbons (MH, λ = −0.08), oxygenated monoterpenes (OM, λ = −0.42), benzenoids/phenylpropanoids (BZ-PP, λ = 0.35), and fatty acids and derivatives (FA, λ = −0.38). Similar to HCA, the PCA analysis confirmed the formation of five distinct groups.

Group I was characterized by oxygenated monoterpene (57.0–67.5%) and monoterpene hydrocarbons (14.8–36.9%). Group II was characterized by oxygenated monoterpenes (36.7–52.4%) and oxygenated sesquiterpenes (20.8–30.4%). Group III was characterized by sesquiterpene hydrocarbons (29.0%), oxygenated sesquiterpenes (26.3%), and benzenoids/phenylpropanoids (21.4%). Group IV was characterized by oxygenated diterpenes (62.0%). Group V was characterized by benzenoids/phenylpropanoids (53.5–59.3%) and oxygenated monoterpenes (24.6–37.6%).

## 3. Materials and Methods

### 3.1. Plant Material

The orchids *Catasetum alatum*, *C. albovirens*, *C. barbatum*, *C. ciliatum*, *C. galeritum*, and *C. gnomus* (Figure 1, Figure 2, Figure 3, Figure 4, Figure 5 and Figure 6), which provided the flowers for this work, are live plants cultivated in pots containing charcoal and wood shavings, existing in the private nursery of Mr. Luiz Otávio Adão Teixeira, located in the Amazon Garden Condominium, BR-316, km 6, 67015-795 Ananindeua, PA, Brazil (coordinates: 1°22′20.96″ S/48°23′34.14′′ W). These orchid specimens were previously sampled in various localities and cities of the Brazilian Amazon, as already described for each one in the Results. Each plant exemplar (exsiccate) was deposited in the Herbarium of Emílio Goeldi Museum, Belém, Para state, Brazil. The orchid flowers were collected during the flowering period, at 6 am, to extract their volatile constituents.

### 3.2. Obtaining and Analyzing Volatile Concentrates

The orchid flowers were subjected to micro distillation-extraction in a Likens & Nickerson-type apparatus (3 flowers each, 15 g in total, 2 h, duplicate) to obtain their volatile concentrates, using *n*-pentane (99% HPLC grade, 3 mL) as the solvent [26].

The volatile concentrates of orchids were submitted to GC and GC-MS analysis. It was performed on a GCMS-QP2010 Ultra system (Shimadzu Corporation, Tokyo, Japan), equipped with an AOC-20i auto-injector and the GCMS-Solution software containing the Adams (2007), Mondello (2011), and Nist (2011) libraries [27,28,29]. A Rxi-5ms (30 m × 0.25 mm; 0.25 μm film thickness) silica capillary column (Restek Corporation, Bellefonte, PA, USA) was used. The conditions of analysis were as follows. Injector temperature: 250 °C; Oven temperature programming: 60–240 °C (3 °C min^−1^); Helium as the carrier gas, adjusted to a linear velocity of 36.5 cm s^−1^ (1.0 mL min^−1^); split mode injection (split ratio 1:20) of 1.0–2.0 µL of the *n*-pentane solution; electron ionization at 70 eV; ionization source and transfer line temperatures of 200 and 250 °C, respectively. The mass spectra were obtained by automatically scanning every 0.3 s, with mass fragments in the 35–400 m/z. The retention index was calculated for all volatile components using a homologous series of C8-C40 *n*-alkanes (Sigma-Aldrich, Milwaukee, WI, USA) according to the linear equation of van den Dool and Kratz (1963) [30]. Individual components were identified by comparing their retention indices and mass spectra (molecular mass and fragmentation pattern) with those existing in the GCMS-Solution system libraries [27,28,29]. The quantitative data regarding the volatile constituents were obtained using a GC2010 Series gas chromatograph, operated under similar conditions to those of the GC-MS system. The relative amounts of individual components were calculated by peak-area normalization using a flame ionization detector (GC-FID). Chromatographic analyses were performed in duplicate.

### 3.3. Multivariate Statistical Analysis

Principal Component Analysis (PCA) was applied to verify the interrelationship of the samples of volatile concentrates analyzed with the classes of identified compounds, monoterpene hydrocarbons (MH), oxygenated monoterpenes (OM), sesquiterpene hydrocarbons (SH), oxygenated sesquiterpenes (OS), oxygenated diterpenes (OD), benzenoids/phenylpropanoids, and fatty acids and derivatives. The data matrix was standardized for multivariate analysis by subtracting the mean and dividing it by the standard deviation. Hierarchical Cluster Analysis (HCA), considering the Euclidean distance and complete linkage, was used to verify the similarity of the samples based on the distribution of the constituents selected in the PCA analysis (Software Minitab, free version 390, Minitab Inc., State College, PA, USA) [31].

## 4. Conclusions

In conclusion, the present study showed that previous reports were not found in the literature concerning the chemotaxonomy of the *Catasetum* species. Thus, considering their classes of compounds, *Catasetum albovirens*, *C. gnomus*, and *C. barbatum* can be distinguished from the other studied species, while *C. alatum*, *C. galeritum*, and *C. ciliatum* showed the same primary compound classes. Also, there are two chemotypes of *C. ciliatum*, the first one rich in oxygenated monoterpene (67.5%) and the second rich in benzenoids/phenylpropanoids (53.5%). Thus, we think these findings could contribute to a better understanding of the chemical profiles of *Catasetum* species.

## Figures and Tables

**Table 1:** Constituents identified in the volatile concentrate of *C. alatum* flowers.

Constituents	RI_Cal_	RI_Lit_	%
**α-Pinene**	**929**	**932 ^a^**	**11.8**
Sabinene	968	969 ^a^	0.1
β-Pinene	975	974 ^a^	0.5
Myrcene	987	988 ^a^	1.3
α-Phellandrene	1002	1002 ^a^	3.6
δ-3-Carene	1008	1008 ^a^	0.3
**Limonene**	**1024**	**1024 ^a^**	**18.2**
(*E*)-β-Ocimene	1043	1044 ^a^	0.2
Bergamal	1049	1051 ^a^	0.1
γ-Terpinene	1054	1054 ^a^	0.2
*cis*-Linalool oxide	1067	1067 ^a^	0.7
*trans*-Linalool oxide	1084	1084 ^a^	0.4
*p*-Cymenene	1089	1089 ^a^	0.2
**Linalool**	**1095**	**1095 ^a^**	**8.8**
1,3,8-*p*-Menthatriene	1108	1108 ^a^	0.2
*cis-p*-Menth-2-en-1-ol	1116	1118 ^b^	0.9
*trans-p*-Mentha-2,8-dien-1-ol	1118	1119 ^a^	0.8
*allo*-Ocimene	1128	1128 ^a^	0.3
*trans-p*-Menth-2-en-1-ol	1135	1136 ^b^	0.6
*trans*-Limonene oxide	1136	1137 ^a^	3.8
*p*-Menta-1,5-dien-8-ol	1164	1166 ^a^	0.2
3,7-dimethyl-(*Z*)-2,6-Octadienal	1173	1174 ^b^	0.3
Terpinen-4-ol	1178	1180 ^b^	0.5
*p*-Cymen-8-ol	1186	1189 ^b^	1.0
dihydro-Carveol	1190	1192 ^a^	0.1
α-Terpineol	1192	1195 ^b^	0.8
*cis*-Piperitol	1194	1195 ^a^	0.6
*trans*-dihydro-Carvone	1204	1200 ^a^	0.2
*trans*-Piperitol	1208	1207 ^a^	0.7
Coahuilensol methyl ether	1215	1219 ^a^	0.1
*trans*-Carveol	1218	1223 ^b^	0.7
*trans*-chrysanthenyl acetate	1227	1231 ^b^	3.9
*cis*-Carveol	1231	1232 ^b^	0.5
Neral	1233	1235 ^a^	0.1
Carvone	1237	1239 ^a^	0.1
**Geraniol**	**1246**	**1249 ^a^**	**29.7**
(2*E*)-Decenal	1258	1260 ^a^	0.4
Geranial	1262	1264 ^a^	0.3
Citronellyl formate	1269	1271 ^a^	0.1
Neryl formate	1279	1280 ^a^	0.1
(4*E*,4*Z*)-Decadienal	1294	1292 ^a^	0.1
Perilla alcohol	1298	1294 ^a^	0.1
*p*-vinyl-Guaiacol	1308	1309 ^a^	0.3
(2*E*,4*E*)-Decadienal	1312	1315 ^a^	0.1
Methyl geranate	1321	1322 ^a^	0.7
(2*E*)-Undecenal	1356	1357 ^a^	0.1
α-Copaene	1374	1374 ^a^	0.1
Geranyl acetate	1378	1379 ^a^	0.2
β-Elemene	1388	1389 ^a^	0.5
β-Longipinene	1400	1400 ^a^	0.1
dihydro-α-Ionona	1411	1411 ^a^	0.1
(*E*)-Caryophyllene	1415	1417 ^a^	0.4
*trans*-α-Bergamotene	1427	1432 ^a^	0.1
α-Humulene	1450	1452 ^a^	0.2
Germacrene D	1479	1480 ^b^	0.1
Bicyclogermacrene	1493	1497 ^b^	0.1
α-Muurolene	1496	1500 ^a^	0.1
*trans*-β-Guaiene	1502	1502 ^a^	0.1
δ-Amorphene	1510	1511 ^a^	0.4
(*E*)-Nerolidol	1560	1561 ^a^	0.5
Germacrene D-4-ol	1574	1574 ^a^	0.1
Hexadecane	1600	1600 ^a^	0.1
*epi*-α-Cadinol	1636	1638 ^a^	0.1
*epi*-α-Muurolol	1638	1640 ^a^	0.1
α-Cadinol	1651	1652 ^a^	0.3
Monoterpene hydrocarbons			36.9
Oxygenated monoterpenes			57.0
Sesquiterpene hydrocarbons			2.2
Oxygenated sesquiterpenes			1.2
Benzenoids/Phenylpropanoids			0.3
Fatty acid and derivatives			0.9
Total (%)			98.5

RI_Cal_ = Calculated Retention Index; RI_Lit_ = Literature Retention Index; ^a^ Adams, 2007; ^b^ Mondello, 2011; Bold = Main constituents. Unidentified minor constituents were 1.5%.

**Table 2:** Constituents identified in the volatile concentrates of two specimens of *C. albovirens* flowers.

Constituents (%)	RI_Cal_	RI_Lit_	Calb-1	Calb-2
α-Pinene	929	932 ^a^	4.7	0.2
Sabinene	968	969 ^a^	0.5	-
β-Pinene	972	974 ^a^	0.8	-
Myrcene	986	988 ^a^	1.6	3.4
δ-3-Carene	1006	1008 ^a^	-	-
α-Terpinene	1011	1014 ^a^	0.1	-
Limonene	1023	1024 ^a^	-	0.2
**1,8-cineole**	1025	1026 ^a^	**11.7**	1.1
(*Z*)-β-Ocimene	1029	1032	-	0.2
(*E*)-β-Ocimene	1043	1044 ^a^	0.6	3.8
Bergamal	1049	1051 ^a^	0.8	1.4
γ-Terpinene	1053	1054 ^a^	0.3	-
Terpinolene	1085	1086 ^a^	0.1	-
**Linalool**	1093	1095 ^a^	**10.0**	**39.5**
*cis*-Rose oxide	1103	1106 ^a^	0.2	0.3
*n*-Nonanal	1104	1107 ^a^	0.4	0.8
(*E*)-4,8-dimethyl-Nona-1,3,7-triene	1112	1113 ^b^	-	0.1
*trans*-Rose oxide	1120	1122 ^a^	0.1	-
*trans*-Limonene oxide	1135	1137 ^a^	-	0.1
Nerol oxide	1150	1152 ^b^	-	0.2
1,4-Dimethoxybenzene	1160	1161 ^a^	0.4	0.1
δ-Terpineol	1162	1162 ^a^	0.3	-
Terpinen-4-ol	1178	1180 ^b^	0.6	
α-Terpineol	1184	1186 ^a^	4.8	1.1
Methyl salicylate	1190	1190 ^a^	0.2	-
*trans*-Carveol	1212	1215 ^a^	1.0	0.5
Neral	1233	1235 ^a^	0.1	-
Carvone	1237	1239 ^a^	-	0.2
Geraniol	1247	1249 ^a^	1.6	1.7
cis-Carvone oxide	1257	1259 ^a^	-	0.5
Geranial	1261	1264 ^a^	0.1	0.1
Indole	1289	1290	0.3	-
*p*-vinyl-Guaiacol	1308	1309 ^a^	0.3	0.2
Methyl anthranilate	1321	1334 ^a^	0.2	-
2,4-Octanediol	1334	1339 ^a^	0.1	-
Citronellyl acetate	1348	1350 ^a^	0.2	0.2
Neryl acetate	1356	1359 ^a^	0.1	-
Geranyl acetate	1378	1379 ^a^	4.2	3.9
9-Decenyl acetate	1398	1399 ^a^	1.0	0.2
Citronellyl propanoate	1442	1444 ^a^	-	1.4
(2*E*)-Dodecen-1-ol	1465	1469 ^a^	3.3	1.6
**7-*epi*-1,2-dehydro-Sesquicineole**	1471	1471 ^a^	**28.3**	**19.3**
Neryl isobutanoate	1488	1490 ^a^	0.2	-
**(*E*,*E*)-α-Farnesene**	1504	1505 ^a^	1.0	**5.2**
Myrac aldehyde	1514	1519 ^a^	0.5	-
Hedycaryol	1542	1546	0.5	-
Elemol	1546	1548 ^a^	0.2	-
(*E*)-Nerolidol	1560	1561 ^a^	0.9	1,5
**Anisyl butyrate**	1566	1569 ^c^	**15.4**	**7.5**
Monoterpene hydrocarbons			8.7	7.9
Oxygenated monoterpenes			36.7	52.4
Sesquiterpene hydrocarbons			1.0	5.2
Oxygenated sesquiterpenes			30.4	20.8
Benzenoids/Phenylpropanoids			16.1	7.6
Fatty acid and derivatives			4.8	2.6
Total (%)			97.7	96.5

RI_Cal_ = Calculated Retention Index; RI_Lit_ = Literature Retention Index; ^a^ Adams, 2007; ^b^ Mondello,2011; ^c^ Nist, 2011. Bold = Main constituents. Unidentified minor constituents were 2.3% and 3.5%, respectively.

**Table 3:** Constituents identified in the volatile concentrate of *C. barbatum* flowers.

Constituents	RI_Cal_	RI_Lit_	%
Hexanal	795	801 ^a^	0.6
2-pentyl-Furan	989	991 ^a^	0.2
1-p-Menthene	1021	1021 ^a^	0.5
Benzene acetaldehyde	1035	1036 ^a^	0.3
(2*E*)-Octen-1-al	1048	1049 ^a^	0.1
Linalool	1092	1095 ^a^	0.1
*n*-Nonanal	1097	1100 ^a^	0.5
Naphthalene	1176	1178 ^b^	0.1
*cis*-carvone oxide	1256	1259 ^a^	0.1
**Indole**	1289	1290 ^a^	**11.3**
**(*E*)-β-Farnesene**	1451	1454 ^b^	**16.4**
(*E*)-Nerolidol	1558	1561 ^a^	0.2
(5*E*,9*E*)-Farnesyl acetone	1911	1913 ^a^	1.9
(*Z*,*Z*)-Geranyl linalool	1957	1960 ^b^	0.5
(*E*,*E*)-Geranyl linalool	2023	2026 ^b^	0.3
*n*-Heneicosane	2100	2100 ^b^	0.1
***trans*-Geranylgeraniol**	2198	2201 ^c^	**61.2**
*n*-Tricosane	2297	2300 ^b^	0.4
2-methyl-tricosane	2365	2365 ^a^	2.2
1-Docosanol	2500	2500 ^c^	0.3
Monoterpene hydrocarbons			0.5
Oxygenated monoterpenes			0.2
Sesquiterpene hydrocarbons			16.4
Oxygenated sesquiterpenes			2.1
Oxygenated diterpenes			62.0
Benzenoids/Phenylpropanoids			11.8
Fatty acid and derivatives			4.3
Total			97.3

RI_Cal_ = Calculated Retention Index; RI_Lit_ = Literature Retention Index; ^a^ Adams, 2007; ^b^ Mondello, 2011; ^c^ Nist, 2011. Bold = Main constituents. Unidentified minor constituents were 2.7%.

**Table 4:** Constituents identified in the volatile concentrates of two specimens of *C. ciliatum* flowers.

Constituents (%)	RICal	RILit	Ccil-1	Ccil-2
Hexanal	795	801 ^a^	-	0.2
*n*-Octane	796	800 ^a^	-	0.2
Heptanal	898	901 ^a^	-	0.4
**α-Pinene**	**929**	**932 ^a^**	**10.5**	**4.8**
Benzaldehyde	953	952 ^a^		0.2
Sabinene	968	969 ^a^	0.3	0.1
β-Pinene	973	974 ^a^	0.9	0.3
Myrcene	987	988 ^a^	0.7	0.2
α-Phellandrene	1003	1002 ^a^	2.2	0.4
*p*-Cymene	1018	1020 ^a^	0.1	0.1
β-Phellandrene	1025	1025 ^a^	-	2.4
**1,8-Cineole**	1027	1026 ^a^	**23.7**	**3.7**
(*E*)-β-Ocimene	1043	1044 ^a^	0.1	0.1
6,7-Epoxymyrcene	1092	1090 ^a^	0.1	-
Linalool	1095	1095 ^a^	0.1	0.1
*n*-Nonanal	1104	1107 ^a^	0.1	0.6
(2*E*,4*E*)-Octadienal	1118	1113 ^b^	0.1	-
*trans*-Limonene oxide	1136	1137 ^a^	2.6	0.1
**Benzyl acetate**	**1166**	**1167 ^b^**	**11.8**	**22.8**
Naphthalene	1175	1178 ^a^	0.1	-
Terpinen-4-ol	1178	1180 ^b^	0.6	-
α-Terpineol	1184	1186 ^a^	0.2	-
*cis*-Dihydro carvone	1996	1991 ^a^	0.4	-
*trans*-Dihydro carvone	1204	1200 ^a^	0.1	-
*cis*-Carveol	1231	1232 ^b^	0.1	-
**Carvone**	**1242**	**1239 ^a^**	**7.8**	**4.4**
**Geraniol**	**1251**	**1249 ^a^**	**16.6**	**5.0**
***cis*-Carvone oxide**	**1262**	**1262 ^b^**	**14.3**	**10.1**
**2-Phenylethyl acetate**	**1261**	**1254 ^a^**	**3.2**	**30.5**
Geranial	1267	1264 ^a^	0.2	0.1
Dihydrocarveol acetate	1311	1306 ^a^	0.3	0.2
Geranyl acetate	1378	1379 ^a^	0.4	0.9
*n*-Tricosane	2294	2300 ^a^	-	0.4
*n*-Tetracosane	2393	2400 ^a^	-	0.2
Behenic alcohol	2448	2456 ^c^	-	5.4
Monoterpene hydrocarbons			14.8	8.4
Oxygenated monoterpenes			67.5	24.6
Benzenoids/Phenylpropanoids			15.0	53.5
Fatty acid and derivatives			0.3	7.4
Total (%)			97.6	93.9

RI_Cal_ = Calculated Retention Index; RI_Lit_ = Literature Retention Index; ^a^ Adams, 2007; ^b^ Mondello, 2011; ^c^ Nist, 2011. Bold = Main constituents. Unidentified minor constituents were 2.4% and 6.1%, respectively.

**Table 5:** Constituents identified in the volatile concentrate of *C. galeritum* flowers.

Constituents	RI_Cal_	RI_Lit_	%
*n*-Hexanol	861	886 ^a^	0.1
Heptanal	902	901 ^a^	0.1
Myrcene	987	988 ^a^	0.4
Limonene	1026	1024 ^a^	0.1
Sylvestrene	1027	1025 ^a^	0.1
(*Z*)-β-Ocimene	1035	1032 ^a^	0.1
(*E*)-β-Ocimene	1043	1044 ^a^	1.6
Terpinolene	1085	1086 ^b^	0.1
**Linalool**	**1097**	**1095 ^a^**	**34.9**
*allo*-Ocimene	1128	1128 ^a^	0.1
**1,4-Dimethoxybenzene**	1160	1161 ^a^	**54.1**
Terpinen-4-ol	1180	1180 ^a^	0.1
α-Terpineol	1195	1195 ^a^	1.4
Nerol	1231	1229 ^a^	0.3
Geraniol	1251	1249 ^a^	0.9
**Indole**	**1289**	**1290 ^a^**	**5.2**
7-*epi*-Sesquithujene	1390	1390 ^a^	0.1
Monoterpene hydrocarbons			2.5
Oxygenated monoterpenes			37.6
Sesquiterpene hydrocarbons			0.1
Benzenoids/Phenylpropanoids			59.3
Fatty acid and derivatives			0.2
Total			99.7

RI_Cal_ = Calculated Retention Index; RI_Lit_ = Literature Retention Index; ^a^ Adams, 2007; ^b^ Mondello, 2011; Bold = Main constituents. Unidentified minor constituents were 0.3%.

**Table 6:** Constituents identified in the volatile concentrate of *C. gnomus* flowers.

Constituents	RI_Cal_	RI_Lit_	%
α-Pinene	929	932 ^a^	2.2
α-Phellandrene	1004	1002 ^a^	1.5
*p*-Cymene	1018	1020 ^a^	1.5
**Limonene**	**1026**	**1024 ^a^**	**7.5**
1,8-Cineole	1027	1026 ^a^	2.7
Methyl benzoate	1090	1088 ^a^	2.2
1,3,8-*p*-Menthatriene	1110	1108 ^a^	0.5
*cis*-*p*-Menth-2-en-1-ol	1119	1118 ^a^	1.2
*trans*-*p*-Mentha-2,8-dien-1-ol	1121	1122 ^a^	2.8
*trans*-Limonene oxide	1136	1137 ^a^	0.2
**Methyl salicylate**	**1190**	**1190 ^a^**	**17.0**
*cis*-Piperitol	1197	1195 ^a^	0.8
*trans*-Piperitol	1208	1207 ^a^	0.8
Indole	1289	1290 ^a^	1.6
δ-Elemene	1336	1335 ^a^	0.4
α-Cubebene	1349	1349 ^a^	0.9
Eugenol	1355	1357 ^a^	0.6
α-Copaene	1374	1374 ^a^	0.1
β-Elemene	1388	1389 ^a^	0.8
(*E*)-Caryophyllene	1417	1417 ^a^	3.6
*trans*-Muurola-3,5-diene	1450	1451 ^a^	0.2
α-Humulene	1453	1452 ^a^	1.3
*trans*-Cadina-1(6)-4-diene	1473	1475 ^a^	0.2
Germacrene D	1479	1480 ^a^	3.8
(*E*)-β-Ionone	1485	1487 ^a^	0.8
δ-Selinene	1491	1492 ^a^	0.2
*epi*-Cubebol	1494	1493 ^a^	0.5
α-Muurolene	1496	1500 ^a^	1.7
Bicyclogermacrene	1497	1500 ^a^	0.7
(*E*,*E*)-α-Farnesene	1508	1505 ^a^	0.8
γ-Cadinene	1514	1513 ^a^	3.3
**δ-Cadinene**	**1524**	**1522 ^a^**	**10.3**
*trans*-Cadina-1,4-diene	1532	1533 ^a^	0.1
α-Cadinene	1537	1537 ^a^	0.6
Germacrene D-4-ol	1574	1574 ^a^	4.3
Caryophyllene oxide	1583	1582 ^a^	0.2
***epi*-α-Muurolol**	**1641**	**1638 ^a^**	**6.9**
α-Muurolol	1646	1645 ^a^	0.9
**Cadin-4-en-10-ol**	**1656**	**1659 ^b^**	**12.1**
(*Z*)-α-trans-Bergamotol	1693	1690 ^a^	0.6
Monoterpene hydrocarbons			13.2
Oxygenated monoterpenes			8.5
Sesquiterpene hydrocarbons			29.0
Oxygenated sesquiterpenes			26.3
Benzenoids/Phenylpropanoids			21.4
Total			98.4

RI_Cal_ = Calculated Retention Index; RI_Lit_ = Literature Retention Index; ^a^ Adams, 2007; ^b^ Mondello, 2011; Bold = Main constituents. Unidentified minor constituents were 1.6%.

**Table 7:** Classes of compounds identified in *Catasetum* specimens used in the multivariate statistical analyses.

Classes of Compounds (%)	Cala	Calb-1	Calb-2	Cbar	Ccil-1	Ccil-2	Cgal	C.gno
Monoterpene hydrocarbons	36.9	8.7	7.9	0.5	14.8	8.4	2.5	13.2
Oxygenated monoterpene	57.0	36.7	52.4	0.2	67.5	24.6	37.6	8.5
Sesquiterpene hydrocarbons	2.2	1.0	5.2	16.4	1.0	-	0.1	29.0
Oxygenated sesquiterpene	1.2	30.4	20.8	2.1	-	-		26.3
Oxygenated diterpenes	-	^-^	-	62.0	-	-	-	-
Benzenoids/Phenylpropanoids	0.3	16.1	7.6	11.8	15.0	53.5	59.3	21.4
Fatty acids and derivatives	0.9	4.8	2.6	4.3	0.3	7.4	21.4	-
Total (%)	98.5	97.7	96.5	97.3	97.6	93.9	99.7	98.4

## Data Availability

Not applicable.
